# Foliar herbivory increases sucrose concentration in bracteal extrafloral nectar of cotton

**DOI:** 10.1371/journal.pone.0258836

**Published:** 2021-10-29

**Authors:** Cody C. Gale, Pierre Lesne, Caroline Wilson, Anjel M. Helms, Charles P-C. Suh, Gregory A. Sword

**Affiliations:** 1 Department of Entomology, Texas A&M University, College Station, Texas, United States of America; 2 Southern Plains Agricultural Research Center, USDA-ARS, College Station, Texas, United States of America; Universidade Federal de Uberlândia, BRAZIL

## Abstract

Cultivated cotton, such as *Gossypium hirsutum* L., produces extrafloral (EF) nectar on leaves (foliar) and reproductive structures (bracteal) as an indirect anti-herbivore defense. In exchange for this carbohydrate-rich substance, predatory insects such as ants protect the plant against herbivorous insects. Some EF nectar-bearing plants respond to herbivory by increasing EF nectar production. For instance, herbivore-free *G*. *hirsutum* produces more bracteal than foliar EF nectar, but increases its foliar EF nectar production in response to herbivory. This study is the first to test for systemically induced changes to the carbohydrate composition of bracteal EF nectar in response to foliar herbivory on *G*. *hirsutum*. We found that foliar herbivory significantly increased the sucrose content of bracteal EF nectar while glucose and fructose remained unchanged. Sucrose content is known to influence ant foraging behavior and previous studies of an herbivore-induced increase to EF nectar caloric content found that it led to increased ant activity on the plant. As a follow-up to our finding, ant recruitment to mock EF nectar solutions that varied in sucrose content was tested in the field. The ants did not exhibit any preference for either solution, potentially because sucrose is a minor carbohydrate component in *G*. *hirsutum* EF nectar: total sugar content was not significantly affected by the increase in sucrose. Nonetheless, our findings raise new questions about cotton’s inducible EF nectar responses to herbivory. Further research is needed to determine whether an herbivore-induced increase in sucrose content is typical of *Gossypium spp*., and whether it constitutes a corollary of systemic sucrose induction, or a potentially adaptive mechanism which enhances ant attraction to the plant

## Introduction

The function of indirect defenses is to recruit predators and/or parasitoids to defend the plant, as opposed to direct defenses such as toxins or trichomes that act on the herbivores themselves [1]. Plants can recruit natural enemies by offering host-location cues and/or food rewards. For example, plants emit characteristic blends of volatile organic compounds (VOCs) following herbivore wounding that are used by predators and parasitoid wasps to locate herbivores (reviewed in [2]). Many plant species also recruit natural enemies by providing an attractive food source in the form of extrafloral (EF) nectar. EF nectar, which is typically rich in sucrose, glucose, and fructose, encourages ants, parasitoids, and other predatory arthropods such as spiders [3, 4] to forage for carbohydrates on the plant (reviewed in [5]). This improves the chance of an encounter between the natural enemies and herbivores such as caterpillars that may be damaging the plant.

Cotton plants, *Gossypium spp*. (Malvales: Malvaceae), use both forms of indirect defense, emitting a bouquet of VOCs following herbivore wounding [6–10] and by bearing EF nectaries on leaves and leaf-like bracts that enclose reproductive structures [11–13]. Cotton produces EF nectar and VOCs constitutively, *i*.*e*., in the absence of herbivory, and augments production as part of an induced response to herbivory that is modulated by jasmonic acid (JA) and JA-related plant hormones [1, 5, 14, 15]. Herbivore-induced VOC emissions are well documented for cotton (*e*.*g*., [6–10]), but EF nectar induction has only been reported for leaf and not bracteal nectaries [11, 12, 16].

In the genus *Gossypium*, at least 36 species are known to bear EF nectaries while only a single species lacks them, *G*. *tomentosum* (Nuttall ex Seeman) [17, 18]. *G*. *tomentosum* is native to Hawaii where there are no native ant species. The lack of EF nectaries on this species lends support to the hypothesis that their maintenance comes with an allocation cost to the plant, and that many *Gossypium* species have maintained EF nectaries through a facultative mutualism with ants [18–20]. Furthermore, in field studies of the wild cotton, *G*. *thurberi* (Todaro), ant exclusion led to significantly higher caterpillar damage, clearly demonstrating the defensive role that ants play [21].

Wäckers & Bonifay [12] hypothesized that, if EF nectar was truly a defensive secretion, even if indirect, patterns of secretion should match predictions from the Optimal Defense Theory (ODT). ODT predicts that individual plants allocate limited defensive resources based on the value of the tissue and the likelihood of herbivore attack, thus optimizing the cost-to-benefit balance of the resource investment [22, 23]. Wäckers and Bonifay [12] tested this hypothesis with *G*. *hirsutum* L. because each true leaf bears a small EF nectary on the mid vein, and each reproductive structure has three larger EF nectaries below the leaf-like bracts that enclose the flower bud and eventually the fruit. These are referred to as foliar and bracteal nectaries, respectively. They found support for the predictions of ODT, observing highest constitutive EF nectar production coupled with a non-inducible response at the bracteal nectaries, and correspondingly lower constitutive, but highly inducible, production at foliar nectaries.

We designed this study to test the hypothesis that bracteal EF nectaries of *G*. *hirsutum* flowers respond systemically to foliar herbivory. We suspect that previous studies did not observe induction at bracteal nectaries because herbivores were placed directly on reproductive structures [12] and cotton tends to reduce investment in damaged fruits [24]. Considering the findings of [25, 26], inducible EF nectar responses to herbivory at reproductive structures might be more typical during flower rather than fruit development. Here, we subjected flowering *G*. *hirsutum* plants to foliar herbivory by caterpillars and compared the carbohydrate composition of bracteal EF nectar before and after herbivore exposure. We quantified sucrose, glucose and fructose, and found that sucrose content increased in response to herbivory. To determine whether this change in carbohydrate composition influenced its effectiveness as an indirect defense by recruiting natural enemies, we also examined ant foraging preferences for EF nectar produced in response to our herbivory treatments.

## Material and methods

### Plant material

Cultivated cotton (*G*. *hirsutum*) variety PHY-367-WRF (PhytoGen Cottonseed, Corteva Agriscience, Wilmington, DE) was used for this study. This variety is genetically engineered to contain insect-killing *Bacillus thuringiensis* (*Bt*) toxins. A total of 190.4 million hectares of genetically engineered crops were grown globally in 2019 [27]. We chose this genetically engineered variety for relevance to contemporary growing practices, and for consistency with concurrent experiments in our lab.

**Fig 1:**
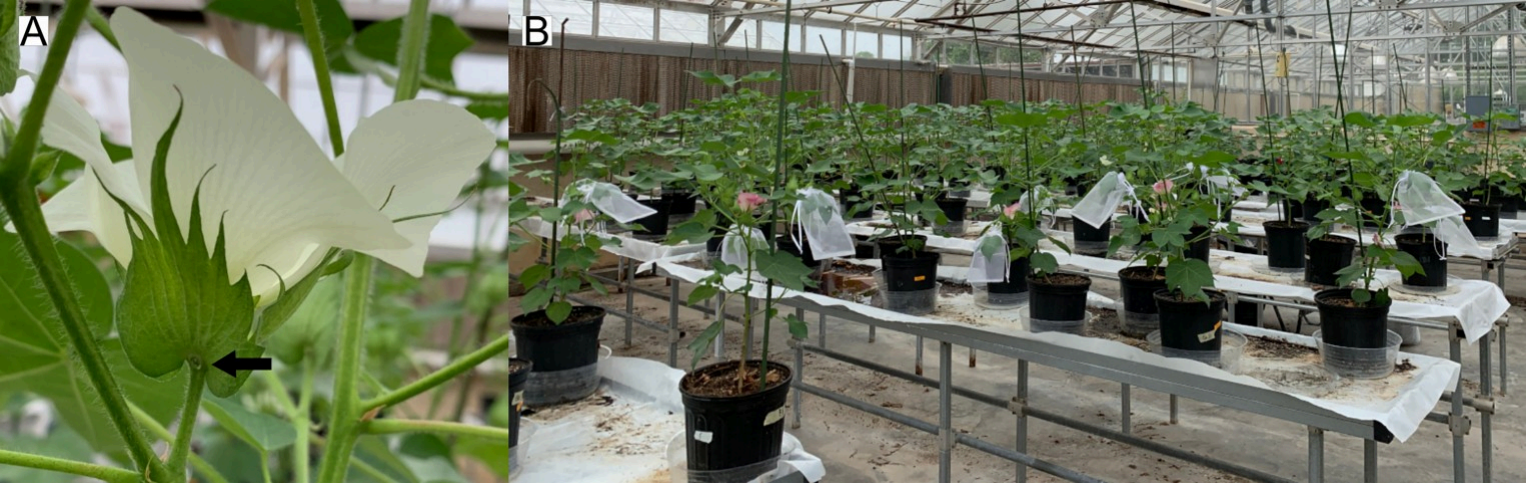
Bracteal EF nectary of *G*. *hirsutum* and the greenhouse during experimentation. (A) *G*. *hirsutum* flower on the day of anthesis. A droplet of nectar can be seen produced by the bracteal EF nectary at the base of one of the three leaf-like bracts that surround the reproductive structure. (B) Plants in the foreground with leaves covered by white organza bags are being exposed to herbivory while plants in the background have not yet reached the day of anthesis for the first flower.

In varieties of *G*. *hirsutum* that bear EF nectaries (some varieties lack EF nectaries, [28]) the underside of each leaf bears a single EF nectary on the midvein (foliar EF nectary), and each of the three leaf-like bracts that enclose the reproductive structures bears an EF nectary at their base (bracteal EF nectary) (Fig 1A). To quantify the carbohydrate composition, at least 1 μL of EF nectar was needed. We found that the individual foliar EF nectaries of the experimental plants did not produce sufficient quantities. In contrast, the three bracteal EF nectaries consistently produced several microliters. Thus, the EF nectar assessed in this study was collected only from bracteal EF nectaries.

### Plant growth conditions

Seeds were planted in 150 cc 6-cell seed-starter trays filled with Jolly Gardener Pro-Line C/25 growing mix (Oldcastle Lawn and Garden, Poland Spring, ME). Plants were grown in environmental chambers (Percival Scientific, Inc., Perry, IA) on a 16:8 (L:D) h cycle and 28:22°C (L:D) for approximately 4 weeks. Experimental plants were then transplanted in the greenhouse into 2-gallon plastic pots filled with the same soil and fertilized with 1 L of 1% v/v CNS Grow 3-1-2 (Botanicare, Vancouver, WA) liquid fertilizer every 4 weeks. Experiments were conducted in a greenhouse (Fig 1B). Plantings were performed regularly, every 1–2 weeks, so that a steady supply of plants bearing their first reproductive structures would be available. The greenhouse was kept insect-free and at approx. 8 weeks plants began to flower.

### Insects

The beet armyworm, *Spodoptera exigua* (Hübner) (Lepidoptera: Noctuidae), was chosen for this experiment because the larvae are primarily foliar feeders, and late-instar larvae can withstand considerable amounts of *Bt-*toxins. Additionally, *S*. *exigua* has been used to induce direct and indirect defensive chemical responses in cotton in numerous studies [29–34]. Furthermore, application of *S*. *exigua* oral secretions systemically induces indirectly defensive VOCs without the need for extensive larval feeding [9].

Eggs of *S*. *exigua* were obtained from Benzon Research Inc. (Carlisle, PA). Upon hatching, larvae were reared individually in 4-cm diameter by 4-cm deep plastic cups on artificial diet (Southland Products Inc., Lake Village, AR) in a rearing room kept at ~28°C on a 14:10 h L:D cycle. Once larvae reached the third instar, they were transferred from the diet cups into glass Petri dishes containing conventional (non-*Bt*) cotton leaves so that they could acclimate to feeding on leaf tissue, and were kept in the rearing room until the start of the experiment.

### Experimental procedures

Cotton flowering follows a predictable developmental pattern, with flowers blooming individually, about 3 days apart, at the same fruiting position of successional branches [24]. This predictable time interval was used to systematize the amount of herbivory to which individual plants were exposed. Peak EF nectar production at bracteal EF nectaries occurs on the day of anthesis, the day a flower blooms [12]. Based on preliminary collections, it was determined that 5 μL of bracteal EF nectar could be consistently collected on the day of anthesis from the first-position flowers on the first two branches.

**Fig 2:**
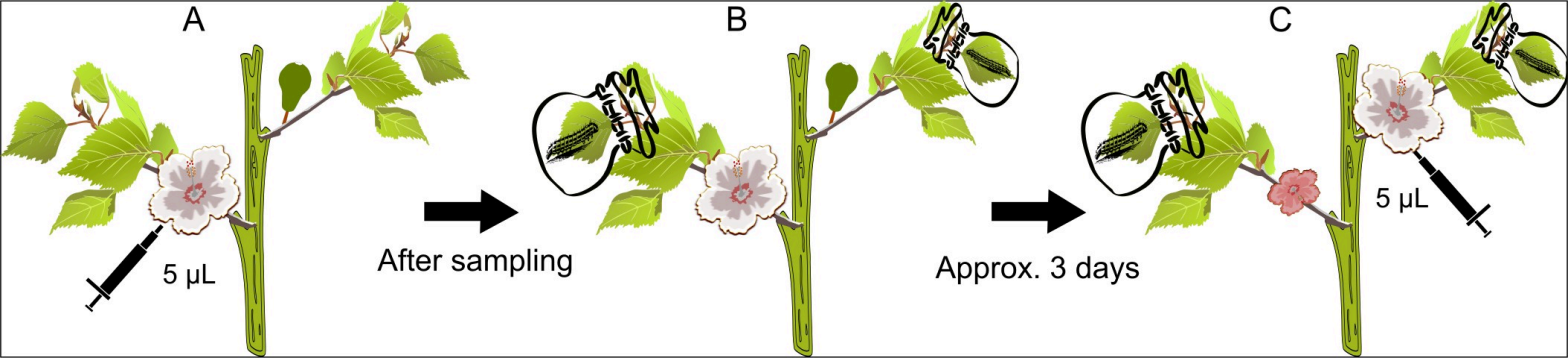
Experimental procedure for collecting constitutive and induced EF nectar samples. (A) A greenhouse plant of *G*. *hirsutum* on the day of anthesis for the first flower. A flower bud develops on the next branch. A total of 5 μL EF nectar is collected from the three bracteal nectaries. This sample, collected prior to plant exposure to herbivory, is the constitutive sample. (B) Immediately following, the terminal leaf of the branch bearing the flower on the day of anthesis is enclosed with two *S*. *exigua* larvae using a draw-string organza bag. The terminal leaf of the next branch, the branch that would bear a flower on the day of anthesis in approx. three days, was enclosed similarly. (C) Another 5 μL of bracteal EF nectar is collected from the second flower. This sample, collected after approx. 3 days of exposure to *S*. *exigua*, is the induced sample. The corolla of the flower from which the constitutive sample was collected has shriveled and turned pink, typical of *G*. *hirsutum* (see Fig 1B for picture).

A total of 46 *G*. *hirsutum* plants were used to test for the effects of herbivory on the carbohydrate composition of bracteal EF nectar. At 1000 h on the day of anthesis for the first flower (day 1 of the experiment), 5 μL EF nectar was collected from the bracteal nectaries, using graduated micropipettes with a metal plunger (Drummond Scientific, Broomall, PA), and dispensed into 45 μL HPLC-grade water (Fig 2A). The samples collected in the absence of herbivory are referred to as the constitutive samples. Immediately following these collections, plants were exposed to herbivory by enclosing the terminal leaf of the blooming branch with two 4^th^- or 5^th^-instar *S*. *exigua* larvae using a draw-string Organza bag (Fig 2B). The same was done to the terminal leaf of the succeeding branch, the branch that would bear a first-position flower in approx. 3 days. On the day of anthesis for the flower of the succeeding branch, another 5 μL EF nectar sample was collected from the bracteal nectaries in the same manner (Fig 2C). The samples collected during exposure to herbivory are referred to as the induced samples.

As mentioned above, EF nectar was collected from bracteal rather than foliar nectaries because of the insufficient volume produced by foliar nectaries. However, to collect herbivore-induced samples, *S*. *exigua* larvae were placed on leaves rather than the flowers because cotton typically sheds or reduces growth of damaged reproductive structures, a process that can halt EF nectar production [12, 24].

A set of control plants, N = 19, were used to test for qualitative differences in the bracteal EF nectar of the first-position flowers of the first two branches in the absence of herbivory. These plants were sampled as described above, except that no *S*. *exigua* larvae were placed on the plants. The purpose of this set of control plants was to determine, in the case that constitutive and induced samples differed, that the variation was due to herbivory and not due to variation in EF nectar produced on different branches.

Samples were frozen at -20°C until the experiments were complete. Immediately before chemical analysis, the samples were thawed at room temperature. The thawed samples were sonicated for 2 minutes to ensure total dissolution of sugars and then passed through 0.4 μm filters [35].

### Chromatographic procedures

Samples were analyzed by high performance liquid chromatography to refractive index detection (HPLC-RID). Sucrose, glucose, and fructose were quantified using standard curves, with pure standards purchased from Sigma-Aldrich (St. Louis, MO). Injections (3 μL) were performed with an autosampler on an HPLC system (1260 Infinity II, Agilent Technologies, Inc., Santa Clara, CA, USA) equipped with a Hi-Plex Calcium ion exchange column (300 mm length, 7.7 mm ID, Agilent Technologies, Inc.). The column compartment was maintained at 80°C and the RID at 55°C. The chromatographic method was isocratic with 100% HPLC-grade water at a flow rate of 0.4 mL/min for 30 min [35].

### Ant recruitment field tests

Given the results of the carbohydrate analysis, field experiments were carried out to assess whether ant recruitment was affected by variation in nectar sucrose concentration. Two artificial stock solutions were made with high-purity sucrose, glucose, and fructose (Sigma-Aldrich, St. Louis, MO) to mimic the average carbohydrate compositions of constitutive and induced EF nectar samples as determined in the greenhouse experiment.

Mock constitutive and induced EF nectar solutions were formulated from the results of the carbohydrate analyses. Only sucrose concentration was allowed to vary. Constitutive and induced formulations both contained 367 mg/mL fructose and 384 mg/mL glucose, while the constitutive formula contained 69 mg/mL sucrose and the induced formula contained 83 mg/mL sucrose.

**Fig 3:**
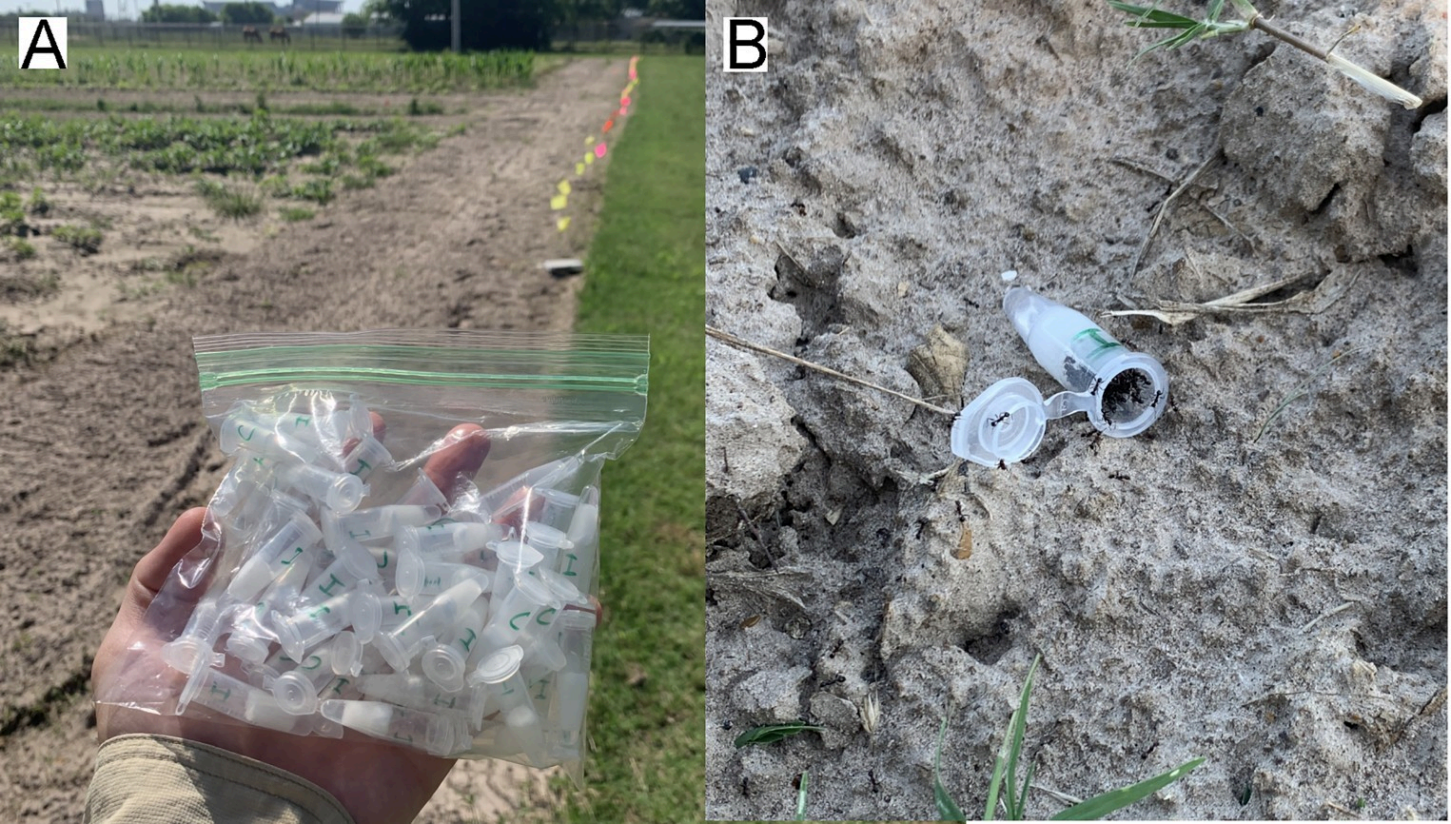
Ant recruitment in the field. (A) Bag of 50 mock EF nectar tubes, 25 mimicking the constitutive composition, labeled with a “C”, and 25 mimicking the induced composition, labeled with an “I”. Colored flags in the background mark the fifty 1 m sections that comprise a transect. The tubes were mixed in the bag and randomly selected for placement along the transect, on the ground to the left of the flags. (B) Close-up of a tube after 1 hr. In this case, a relatively large number of *Pheidole obscurithorax* were recruited.

Five hundred tubes of artificial nectar were prepared by stuffing 1.5 mL microcentrifuge tubes with 100 mg of cotton fiber saturated with 1 mL of either nectar formulation and snapping the lid shut. Tubes were combined in batches of 50, 25 of each nectar formula, and mixed in plastic bags (Fig 3A).

Two sites located near the Texas A&M campus were selected: the test at site 1, a managed grass lawn (30.48039, -96.24539), took place on June 15, 2020; the test at site 2, the edge of a small agricultural field (30.61466, -96.36313), took place on August 1, 2020. One hour before sunset on each site, 5 linear transects were laid out, each 50 m in length and separated from one another by 25 m (Fig 3A). At every meter along each transect, one tube was randomly selected from the plastic bag, opened, and placed on the ground (Fig 3B). A total of 250 tubes were placed at each site. After one hour, tubes were snapped shut and collected, enclosing ants recruited to the nectar [36]. All tubes were frozen at -20°C to kill ants for subsequent counting and identification in the laboratory.

### Statistical analyses

#### EF nectar carbohydrate composition

All analyses were performed in RStudio with R version 3.6.3 [37]. The monosaccharides, glucose and fructose (hexoses), in EF nectar are the result of post-secretory hydrolysis of the disaccharide sucrose by invertase enzymes [38]. Due to the shared origin of glucose and fructose (*i*.*e*., non-independent measurements), the variables are analyzed separately as sucrose, the sum of the hexose concentrations (glucose + fructose), and total sugar content. Quantities were square-root transformed to meet assumptions of normality.

To test for the effects of herbivory (N = 46) on sucrose, hexose, and total sugar content, repeated measures ANOVA was carried out. These data are the S1 Dataset and the R code for statistical analysis is the S1 Script. To test for positional differences between the first-position flowers on neighboring branches of non-treated plants in the absence of herbivory (N = 19), one-way repeated measures ANOVA was performed. These data are the S2 Dataset and the R code for statistical analysis is the S2 Script. Residuals were tested for normality using Shapiro-Wilk’s test and homogeneity of variance tested with Levene’s test from the “car” package (version 3.0–7) [39].

#### Ant recruitment

Ant recruitment to constitutive and induced EF nectar formulations were compared two ways: the percentage of vials in each transect that contained one ant or more (hit percent, [36]) and the average number of ants recruited to vials that had at least one ant (empty vials are not included in the computation of the average recruitment). Each transect constitutes the unit of replication, and N = 10 for both groups.

To test for differences in hit percent, a generalized linear mixed effect model (GLMM) with a binomial distribution was carried out in R using the “lme4” package (version 1.1–23) [40]. Hits were treated as a Bernouilli response variable (0: no ants, 1: at least one ant), formulation (constitutive or induced) as the fixed effect, and transect nested within site as the random effect.

To test for differences in the number of ants recruited, a GLMM with a zero-truncated negative binomial distribution (from “glmmTMB” package version 1.0.2.1, [41]) was used because no zeroes (*i*.*e*., only vials containing ants) were included in the analysis. This model took the same form as above, with ant count as the response variable, formulation (constitutive or induced) as the fixed effect, and transect nested within site as the random effect. Model diagnostics were performed with the “DHARMa” package (version 0.3.3.0) [42]. These data are the S3 Dataset and the R code for statistical analysis is the S3 Script.

## Results

### EF nectar carbohydrate composition

The carbohydrate composition of the EF nectar in our study was similar to that previously reported for cotton [12], with the monosaccharides glucose and fructose as the dominant components at similar concentrations and sucrose as the minor component. Overall, the nectar in our study contained an average of 827 ± 31 mg/mL total sugar (mean ± 1 SE). The carbohydrate composition was dominated by fructose and glucose at mean concentrations of 367 mg/mL and 384 mg/mL, respectively. Sucrose was a much less abundant component, approx. 5 times lower in concentration with an overall mean of 76 mg/mL.

**Fig 4:**
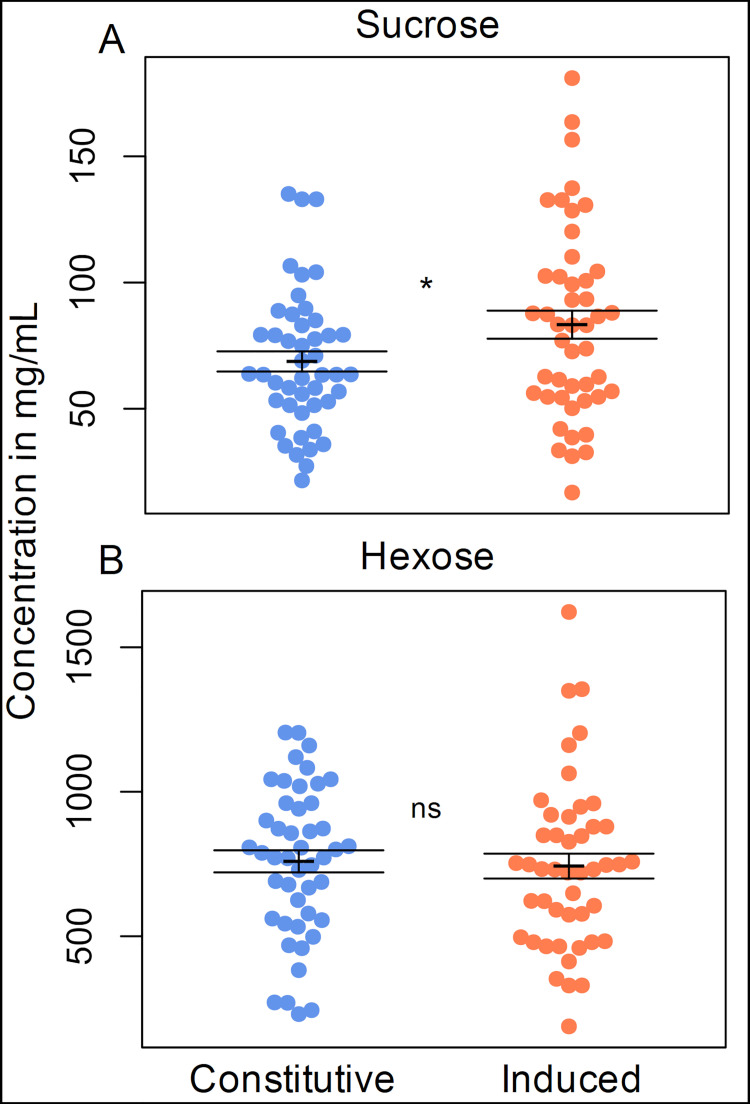
Carbohydrate composition of *G*. *hirsutum* EF nectar. (A) Sucrose and (B) Hexose (total glucose and fructose) concentrations in bracteal EF nectar when herbivores were absent (constitutive) or present (induced). Sucrose concentration increased from an average of 69 mg/mL in constitutive samples to 83 mg/mL in induced samples. Means ± 1 SE shown with black brackets. * = significantly different, ns = not significantly different at α = 0.05.

The repeated measures ANOVA revealed that sucrose concentration was significantly altered by herbivory. Sucrose concentration increased from an average of 69 mg/mL in constitutive samples to 83 mg/mL in induced samples (Fig 4A). Hexose (total glucose and fructose) concentration was unaffected (Fig 4B). The increase in sucrose concentration alone did not significantly increase total sugar content, as sucrose is the least abundant form of sugar (Table 1).

**Table 1:** Analysis of variance table: Repeated measures ANOVA test for effects of herbivory. Sucrose, hexose, and total sugar content of *G*. *hirsutum* EF nectar was compared before and after herbivore exposure.

Response	Variation	Source	df	SS	MS	*F*	P-value
Sucrose	Between plant	Error	45	271.80	4.84		
	Within plant	Herbivory	1	13.26	13.26	6.142	0.017*
		Error	45	97.14	2.16		
Hexose	Between plant	Error	45	1638	36.40		
	Within plant	Herbivory	1	2.80	2.84	0.158	0.693
		Error	45	807.60	17.95		
Total Sugars	Between plant	Error	45	1726	38.35		
	Within plant	Herbivory	1	0.10	0.10	0.005	0.942
		Error	45	854.00	18.98		

* = statistically significant at α = 0.05

The one-way repeated measures ANOVA showed no significant differences in the sucrose, hexose, or total sugar concentration of the first-position flowers on neighboring branches in the absence of herbivory (Table 2). This result confirmed that the increase in sucrose reported above was not a result of sampling different branches to collect constitutive and induced samples.

**Table 2:** Analysis of variance table: Repeated measures ANOVA test for first-position flowers on neighboring branches. Bracteal EF nectar of non-treated *G*. *hirsutum* control plants was sampled in the absence of herbivory to determine if differences existed between the flowers on neighboring branches (Branch No.).

Response	Variation	Source	df	SS	MS	*F*	P-value
Sucrose	Between plant	Error	1	4.25	4.25		
	Within plant	Branch No.	1	1.68	1.68	1.294	0.263
		Error	35	45.50	1.30		
Hexose	Between plant	Error	1	0.59	0.59		
	Within plant	Branch No.	1	16.90	16.85	1.322	0.258
		Error	35	446.30	12.75		
Total sugars	Between plant	Error	1	0.06	0.06		
	Within plant	Branch No.	1	18.50	18.48	1.416	0.242
		Error	35	456.80	13.05		

### Ant recruitment field tests

A total of 1764 ants belonging to 7 genera were collected. Among the 608 ants collected on the test site #1 (*i*.*e*., a managed grassland), 482 (79.3%) were *Solenopsis invicta*, 58 (9.5%) were *Pheidole obscurithorax*, 45 (7.4%) were *Dorymyrmex bureni*, 20 (3.3%) were *Nylanderia sp*. and 3 (0.5%) were *Forelius pruinosis*. On site #2 (*i*.*e*., a small agricultural field), 1156 ants were collected among which 549 (47.5%) were *Monomorium minimum*, 470 (40.7%) were *Solenopsis invicta*, 88 (7.6%) were *Pheidole obscurithorax*, 21 (1.8%) were *Forelius pruinosis*, 18 (1.6%) were *Nylanderia sp*., 7 (0.6%) were *Dorymyrmex bureni* and 3 (0.3%) were *Brachymyrmex patagonicus*.

Albeit beyond the scope of this study, statistics on genus specific preferences were computed but no influence of EF nectar formulation was detected. Only the statistics for the ant community as a whole are reported as it constitutes our main hypothesis.

**Fig 5:**
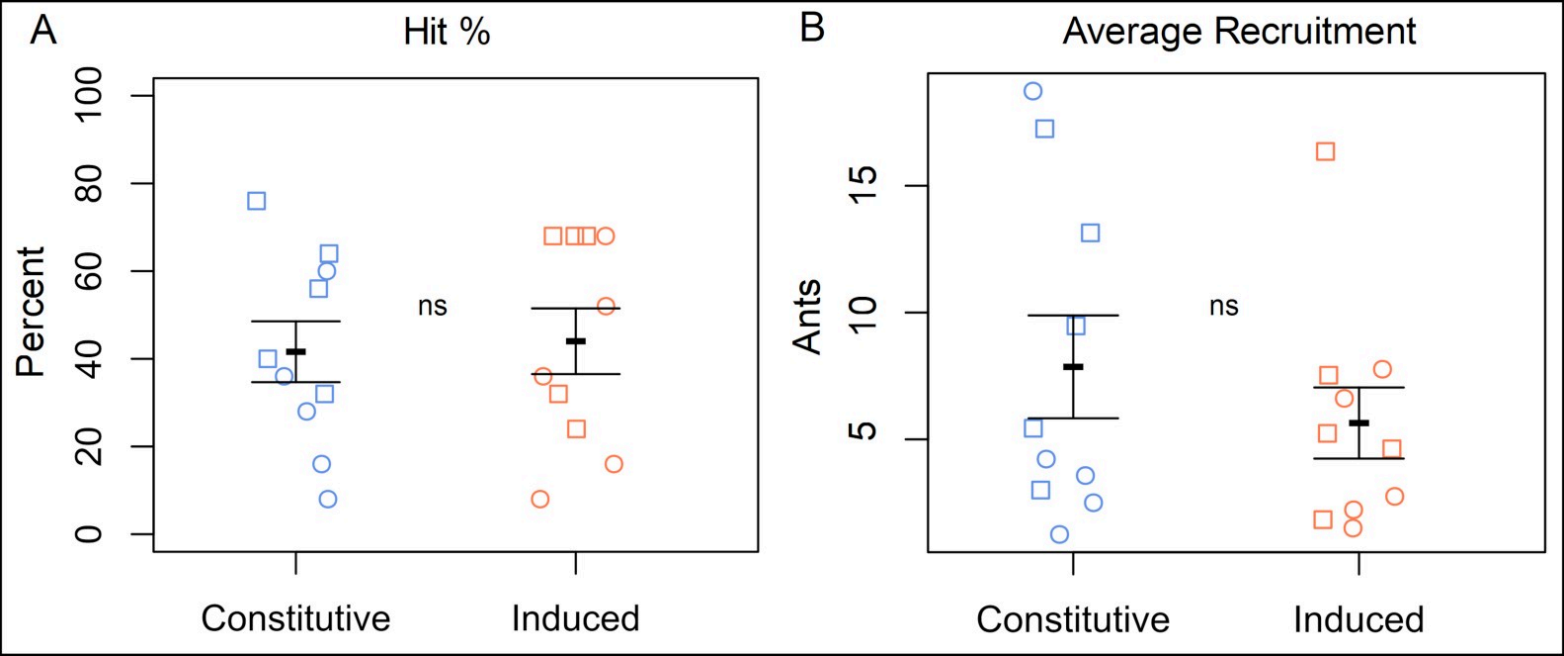
Ant recruitment in the field. (A) Proportion of tubes to which ants were successfully recruited in each transect (B) The mean number of ants per transect in tubes containing at least one ant. Squares are transects at site 1, circles are transects at site 2. Means ± 1 standard error shown with black brackets, ns = not significantly different at α = 0.05.

The proportions of tubes with and without ants (hit percent) were not significantly different between EF nectar formulations (GLMM comparison of the full and null models by Type II Wald Chi-Square test; χ^2^ = 0.352, df = 1, P = 0.552) (Fig 5A), nor was the difference in the number of ants recruited to tubes hit (χ^2^ = 1.66, df = 1, P = 0.198) (Fig 5B).

## Discussion

Our findings revealed a response to foliar herbivory in the sucrose concentration of bracteal EF nectar of *G*. *hirsutum* flowers. When leaves were fed on by caterpillars, the sucrose concentration increased significantly in the bracteal EF nectar produced on the day of anthesis while the hexose concentration was unaffected (Fig 4). Other studies that have examined carbohydrate composition of *G*. *hirsutum* EF nectar in response to herbivory found no changes [11, 12]. Wäckers and Bonifay [12] found that foliar herbivory increased foliar EF nectar production, but they did not test for a systemic response at bracteal EF nectaries. Specifically, they examined foliar EF nectar after foliar herbivory, and bracteal EF nectar after frugivory (*i*.*e*., herbivory on the fruit). Our study thus complements theirs in that we examined bracteal EF nectar after foliar herbivory. Our results also corroborate those of [25, 26] which found that the bracteal EF nectaries at reproductive structures prior to or during flowering respond to herbivory, lending support to the hypothesis that plant strategy for defensive EF nectar investment varies with the age of the structure. Furthermore, our results demonstrate that the bracteal EF nectaries of *G*. *hirsutum* can respond systemically to herbivory elsewhere on the plant.

Our observation of an increase in sucrose concentration might be explained by an increase in JA-dependent cell wall invertase (CWIN) activity. CWIN is central in the redistribution of carbohydrates in response to herbivore damage and is believed to be involved in the unloading of sucrose from phloem to EF nectar [5]. CWIN activity has been shown to be JA-dependent [43], and although we did not measure JA in our experimental plants, caterpillar herbivory is well known to induce JA-dependent responses in *G*. *hirsutum* [44]. Furthermore, in addition to being JA-responsive, CWIN activity is believed to depend on sucrose concentration in the phloem [43], and previous studies have demonstrated that herbivory can induce an increase in the sucrose of aboveground tissues of *Gossypium* [44, 45]. It follows logically that our observation of increased sucrose content in EF nectar could be the product of systemically induced CWIN activity triggered by the rise of JA and phloem sucrose concentration in response to herbivory.

The sucrose concentration of EF nectar has been reported to be a determining factor in the foraging behavior of generalist ants [38]. Invertase enzymes are responsible for the transfer of sucrose from phloem to EF nectar during secretion, and are also responsible for post-secretory hydrolysis of sucrose into glucose and fructose [46]. Generalist ants typically prefer nectars that contain higher sucrose concentrations, as opposed to specialized species involved in ant-plant symbioses (*e*.*g*., *Acacia* trees and *Pseudomyrmex* ants) that prefer low sucrose and/or high monosaccharide concentrations due to their reduced or lack of invertase activity [38]. We hypothesized that the increased sucrose concentration we observed could be a defensive response of the plant to produce EF nectar that is qualitatively more preferable to generalist ants when faced with herbivory.

We found no evidence of a preference for the mock EF nectar with greater sucrose content. We suspect the contribution of several factors detailed below: first, the influence of sugar concentrations in EF nectar and the overall volume of nectar; second, the lack of amino-acids in our mock nectars. The first potential explanation for why we did not observe an ant preference for the nectar solution with more sucrose is that the increase in sucrose concentration observed in our study was not sufficient to significantly alter the total sugar content (Table 1). Total sugar content, which can include sucrose, is reported to be a determining factor in changes to ant foraging preferences to different EF nectars [47, 48]. Typically, damage induces greater amounts of EF nectar produced, making more sugars available to foraging ants even if the concentration of sugars remains the same (*e*.*g*., [3, 11, 12, 26, 49].

In populations of the wild cotton *G*. *thurberi*, ant-plant interactions, EF nectar production, and ant attraction to EF nectar were found to vary geographically [50]. The population of *G*. *thurberi* that produced the highest volume of EF nectar with the highest total sugar concentration received the greatest anti-herbivore benefits from ant visitation. Furthermore, the EF nectar of that particular population contained the highest proportion of sucrose [50]. Those findings lend support to the hypothesis that sucrose concentration is an important factor shaping cotton plant-ant interactions, and that the mock EF nectar formulations in our study may not have been sufficiently distinct to elicit an ant response. *G*. *thurberi* and its interaction with *F*. *pruinosis* ants [51] might thus provide a better study system for similar studies. One species of *Pseudomyrmex* (*P*. *pallidus*) has been found to forage on the EF nectar of *G*. *thurberi* [51] and it would be interesting to test the attraction of this particular species to our mock nectars as well.

Another possible reason for the absence of ant preference between our mock EF nectar solutions could be the lack of amino acids, a minor but important component of EF nectar that we did not measure in this study [5, 46, 52–54]. A response to herbivory in EF nectar chemistry can be an increase in amino acid content. For example, [55] found amino acid concentrations in EF nectar from *Impatiens sultanii* plants increased dramatically in response to foliar herbivory and this was coupled with no change in sugar concentrations. In a follow-up study to the finding of amino acid induction, [54] found that amino acid concentration strongly influenced the feeding preference of fire ants (*Solenopsis spp*.) and, contrary to studies mentioned above, these ants preferred EF nectar that was the least-viscous with the least-concentrated sugars. A previous study found that ant recruitment was highest to mock EF nectar solutions that contained aminos and sugars compared to solutions containing either alone [52]. These studies suggest that the induction of sucrose may be accompanied by amino acids, which in turn may elicit differential ant preference, and this should be investigated.

Previous studies have examined how EF nectar production changes at the site of herbivory, but few have tested for systemically induced changes. While inducible increases in EF nectar production appear to be common, especially on foliar structures [5, 21], reports of increased sugar content in herbivore-induced EF nectar are rare. However, in a recent study that examined *Qualea multiflora* (Mart.), the authors found sugar content increased in addition to increased volume, such that the plant produced a greater volume of more concentrated EF nectar in response to simulated herbivory [25]. Furthermore, the authors observed this effect on reproductive structures, challenging the prediction from ODT that defenses of high-value tissues are non-inducible [12, 22, 23]. Notably, the reproductive structures that they examined were at the flowering, rather than fruiting, stage of development [25]. Similarly, Holland et al. [26] found that EF nectar production was inducible at floral buds of *Pachycereus schottii* (Engelmann) but not at fruits, suggesting that the plant might alter defensive strategies with the age of the structure. Plant defensive strategies for leaves are known to vary with leaf age, and EF nectar production is typically highest and most inducible on young leaves [5, 56]. A similar dynamic defensive strategy might exist for indirect defense at reproductive structures as well. Our results lend support to that hypothesis, considering that previous experiments found a lack of response at *G*. *hirsutum* fruits [12], while we observed an induced response with *G*. *hirsutum* flowers.

Finally, the observed increase in sucrose concentrations could be unrelated to the relationship between EF nectar and ants. Sucrose is the primary form of carbohydrate transport in many plants as it is synthesized in leaves (sources) and reallocated to other tissues such as roots and reproductive organs (sinks) [57]. Sucrose acts as a carbon source that the plant can use to synthesize defensive secondary metabolites, and previous studies of cotton’s many responses to herbivory have reported systemic induction of sucrose in leaves [44, 45]. Because cotton reproductive structures represent a rather strong sucrose sink [58], it is possible that the vasculature in cotton reproductive structures releases additional sucrose into EF nectar when systemic induction of sucrose occurs in response to caterpillar herbivory [44, 59]. In summary, although we demonstrated that foliar herbivory can systemically induce a change in bracteal EF nectar, further examination is needed to understand if the increased sucrose content is ecologically relevant to the relationship that generalist ants have with *G*. *hirsutum*.

## Supporting information

S1 Dataset Sucrose, glucose, and fructose concentrations in extrafloral (EF) nectar of cotton plants exposed to herbivores. Concentrations are in mg/mL. The first column, “Identifier”, provides the ID number assigned to the plant sampled. The “Status” column describes whether the sample was taken before herbivory (CON, short for “constitutive”) or after approx. 3 days of herbivory (IND, short for “induced”). Each plant was sampled twice, before and after herbivory, thus each ID number in the Identifier column appears twice.
(CSV)

S2 Dataset Sucrose, glucose, and fructose concentrations in the extrafloral nectar of cotton plants that were not exposed to herbivory. Concentrations are in mg/mL. These samples were collected to determine whether EF nectar collected from the first flower produced by the plant differed from the EF nectar collected from the second flower produced by the plant. The column “Identifier” again provides the ID number assigned to each plant. The “Flower” column describes which flower was sampled.
(CSV)

S3 Dataset Numbers of ants collected in the field. “Site” provides the site number and “Transect” provides the transect number. “Treatment” describes which artificial formulation the tube contained: “Constitutive” which mimicked the EF nectar of plants before fed on by herbivores, or “Induced” which mimicked the EF nectar of plants after being fed on by herbivores. 25 tubes of each treatment were used at each transect. “Count” provides the number of ants foraging at that tube 1 hr after the tube was placed at the transect.
(TXT)

S1 Script R code used for statistical analysis of S1 Dataset. (R)

S2 Script R code used for statistical analysis of S2 Dataset. (R)

S3 Script R code used for statistical analysis of S3 Dataset. (R)
